# Akt/p27^kip1^ Pathway Is Not Involved in Human Insulinoma Tumorigenesis

**DOI:** 10.1155/2018/7865072

**Published:** 2018-04-26

**Authors:** Adriana Graciela Díaz, Andrea Paes de Lima, Paula Garibaldi, Maria de los Milagros Rubio, Florencia García, Marta Kral, Oscar D. Bruno

**Affiliations:** ^1^Division of Endocrinology, Hospital de Clínicas, Universidad de Buenos Aires, Buenos Aires, Argentina; ^2^Department of Pathology, Hospital de Clínicas, Universidad de Buenos Aires, Buenos Aires, Argentina; ^3^Foundation of Endocrinology (FUNDAENDO), Buenos Aires, Argentina

## Abstract

Insulinomas are pancreatic neuroendocrine tumors (pNET), usually benign. Akt/p27^kip1^ is an intracellular pathway overexpressed in many pNET. There are no data regarding its expression in human insulinomas. We aimed to investigate the expression of Akt and p27^kip1^ in 24 human insulinomas and to compare them to their expression in normal surrounding islets. Staining was performed on embedded paraffin tissue using polyclonal antibodies against total Akt, p-Akt, p27^kip1^, and pp27^kip1^. p-Akt was the predominant form in insulinomas; they presented lower Akt and p-Akt expression than normal islets in 83.3% and 87.5% of tumors, respectively. p27^kip1^ and pp27^kip1^ were mainly cytoplasmic in both insulinomas and normal tissue. Cytoplasmic pp27^kip1^ staining was higher in insulinomas and surprisingly nearly half of the insulinomas also presented nuclear p27^kip1^ (*p* = 0.029). No differences were observed in the subcellular localization of p27^kip1^ and activation of Akt between benign and malignant insulinomas. The low expression of Akt seen in insulinomas might explain the usual benign behavior of this type of pNET. Cytoplasmic p27^kip1^ in both insulinomas and normal islet cells could reflect the low rate of replication of beta cells, while nuclear p27^kip1^ would seem to indicate stabilization and nuclear anchoring of the cyclin D-Cdk4 complex. Our data seem to suggest that the Akt pathway is not involved in human insulinoma tumorigenesis.

## 1. Introduction

Insulinomas are rare pancreatic neuroendocrine tumors (pNET), which arise from islet beta cells. Their incidence is very low, estimated in 1–4 cases per million, and their main clinical characteristic is that they present with hypoglycemic episodes. Most insulinomas occur sporadically or, less frequently, as part of familial cancer syndromes, including multiple endocrine neoplasia type 1 (MEN1), von Hippel Lindau syndrome, neurofibromatosis, and tuberous sclerosis complex [1].

Unlike other pNET, such as gastrinoma or somatostatinoma, insulinomas are usually benign, so it is possible to speculate that this peculiar behavior could be attributed to particular oncogenic events.

Different *in vitro* and *in vivo* animal model studies have brought evidence of the involvement of insulin-like growth factor 2 (IGF2) in insulinoma tumorigenesis [2–4]. By binding and activating the IGF1 receptor (a tyrosine kinase receptor), IGF2 triggers two main downstream pathways: the MAPK and the PI3K/Akt. Both pathways act on p27^kip1^: MAPK induces p27^kip1^ loss, while Akt leads to its mislocalization through the cytoplasm [5, 6].

Akt activity is regulated by different mechanisms that involve membrane translocation and activation by phosphorylation through PI3K, while PTEN hydrolyzes PI3K resulting in Akt inactivation signaling. Akt phosphorylation plays a role in many cellular processes such as cell migration, proliferation, and apoptosis [7]. There is abundant evidence of its upregulation in multiple types of cancer, including neuroendocrine tumors [8, 9].

p27^kip1^ is an important member of the Cip/Kip family of proteins that has a dual activity [10, 11]. In the nucleus, p27^kip1^ acts through binding and regulating the activity of Cdk4, cyclin E/Cdk2, and the cyclin A/Cdk2 complex [12]. When p27^kip1^ is localized in the cytoplasm, Cdk2 is no longer inhibited and it is free to activate E2F1, resulting in cell cycle progression and tumorigenesis, [5, 6, 13]. Recently, there have been reports of other effects of cytoplasmic p27^kip1^ to control cell motility by inhibiting the RhoA-ROCK-LIMK pathway, which has been associated with cancer invasion and metastasis [14]. However, there is evidence that in some tissues, cytoplasmic p27^kip1^ could reduce cell migration and invasion by inhibiting stathmin, a microtubule-destabilizing protein [15–17].

A previous study in MEN1 mutant mice showed a reduction of p27^kip1^ protein expression in 77% of insulinomas [18]. There is no data of Akt and p27^kip1^expression in human insulinomas. The aim of our study was to analyze the expression of Akt and p27^kip1^ in a series of human insulinomas and their surrounding normal tissues to further investigate the role of the Akt/p27 pathway in insulinoma tumorigenesis.

## 2. Subjects and Methods

Twenty-four human pancreatic insulinomas and adjacent normal tissue were obtained from adult patients who had undergone partial pancreatectomy in our institution between 2000 and 2012. Tumors and normal surrounding tissues from the same patient were fixed in formalin and embedded in paraffin blocks after surgery. Pathology confirmed the diagnosis of insulinoma. Twenty-four patients (18 women and 6 men) were enrolled in the current study. Patients' ages ranged from 23 to 88 years (49.5 ± 19.5). Twenty-two of them presented sporadic insulinoma and 2 had MEN 1. Tumor size was 19.27 ± 8.4 mm. Twenty insulinomas were benign and four were malignant.

The study was approved by the local academic and ethics committee (Institutional Review Board) of the Hospital de Clínicas, University of Buenos Aires, according to the Declaration of Helsinki.

The expression of Akt and p27proteins in human insulinomas and their surrounding normal pancreas was examined. Slides of paraffin blocks were incubated with primary antibodies from Santa Cruz Biotechnology Inc., at different dilutions: Akt 1/2/3 (H-136) (total Akt) (RRID: AB 671714) at 1 : 75, phosphoS473Akt 1/2/3 (p-Akt) (RRID: AB 2225021) at 1 : 25, p27 (C-19) (p27^kip1^) (RRID: AB 632129) at 1 : 200, and from Abcam: phosphoThr187-p27 (pp27) (RRID: AB 1310531) at 1 : 25. Bound antibodies were detected using the standard avidin-biotin complex immunoperoxidase system ABC Kit Vectastain Universal from Vector Laboratories Inc. Staining in normal and tumoral tissues was done in parallel for each antibody.

The intensity of cell labeling was ranked using an arbitrary scale: negative (−), low (+), moderate (++), or strong (+++), and the observations were made by two blinded pathologists.

Statistical analysis was performed using SPSS 20.0.

## 3. Results

### 3.1. Expression of Akt in Human Pancreatic Insulinoma and Normal Pancreas

**Figure 1:**
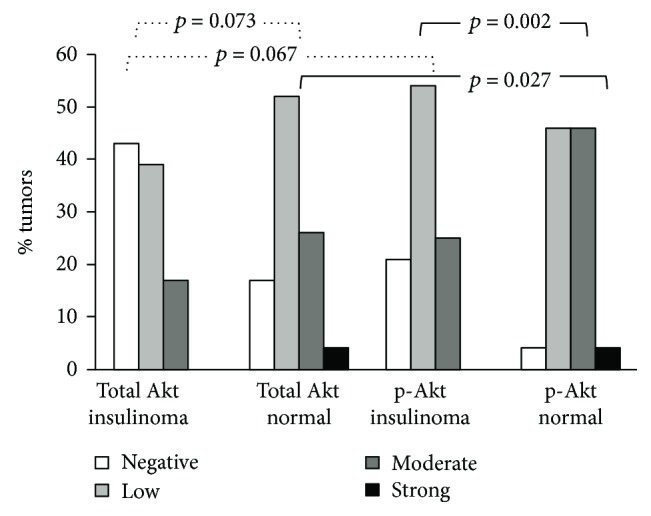
Immunohistochemical expression of Akt in human insulinomas and normal pancreas. Insulinomas presented lower expression of total Akt and p-Akt than normal islets (*p* = 0.002). However, p-Akt was the predominant form in most insulinomas.

**Figure 2:**
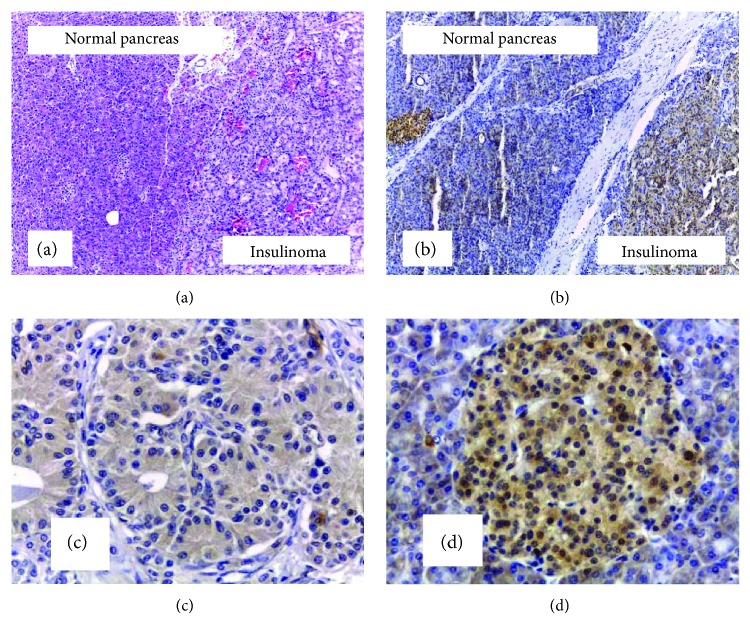
Slides of paraffin blocks of insulinoma and surrounding normal pancreas. (a) Hematoxylin-eosin staining (200x), (b) comparative immunostaining of p-Akt in insulinoma and normal pancreatic islet (200x), (c) immunostaining of p-Akt in insulinoma (400x), and (d) immunostaining of p-Akt in normal islet (400x).

**Figure 3:**
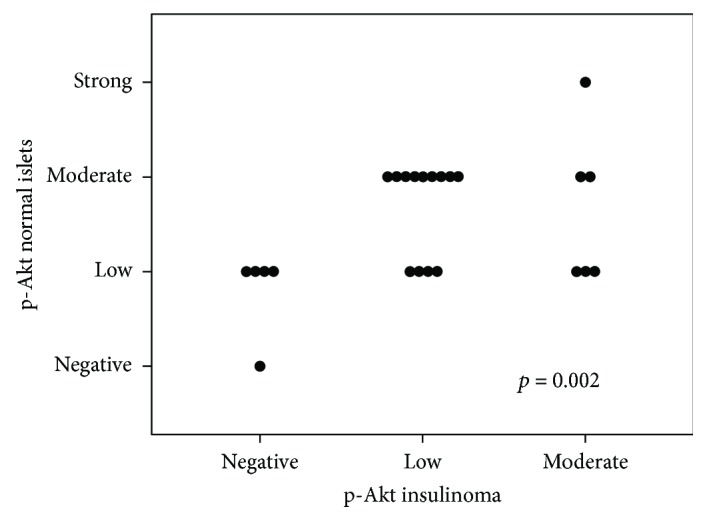
Comparative immunoexpression of p-Akt in normal pancreatic islets and insulinoma. Scatter plot including results of each individual patient showing higher significant expression of p-Akt in normal islets than in insulinoma cells (*p* = 0.002).

Total Akt and p-Akt proteins were detectable in the cellular cytoplasm with a heterogeneous staining pattern. Normal islet cells showed a significant higher cytoplasmic expression of p-Akt than total Akt (Wilcoxon signed-rank test, *p* = 0.027). Similarly, insulinoma cells presented higher expression of p-Akt than total Akt (Wilcoxon signed-rank test, *p* = 0.067), showing the activation of this pathway in both types of cells (Figure 1). However, when we compared the expression patterns of total Akt and p-Akt proteins in human insulinomas with their respective normal surrounding tissues, we found that normal islet cells showed higher or similar expression of total Akt than insulinoma cells (20/24, 83.3%) (Wilcoxon signed-rank test, *p* = 0.073); surprisingly, insulinoma cells showed markedly lower p-Akt staining than their surrounding normal islet cells in 21/24 tumors (87.5%) (Wilcoxon signed-rank test, *p* = 0.002) (Figures 2(b)–2(d) and 3).

### 3.2. Expression of p27^kip1^ in Human Pancreatic Insulinoma and Normal Pancreas

**Figure 4:**
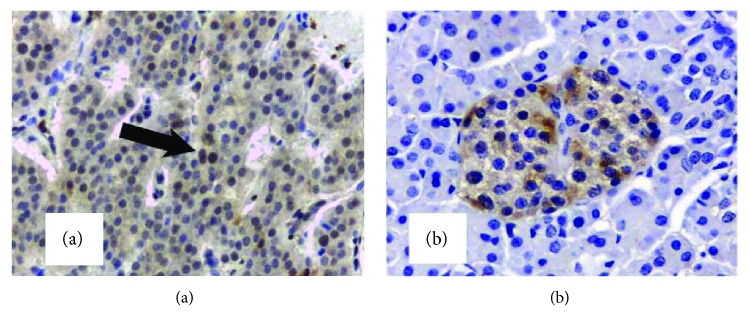
p27^kip1^ presents predominantly cytoplasm localization in human insulinoma (a) and normal pancreatic islet (b); however, insulinoma cells showed higher nuclear expression (arrow) (Wilcoxon signed-rank test, *p* = 0.029) than normal pancreatic islet (400x).

Because of the observed heterogeneity of expression patterns of p27^kip1^ staining localization, we analyzed both nuclear and cytoplasmic p27^kip1^ immunoreactivities. Our studies demonstrated marked levels of p27^kip1^ protein in the nucleus and cytosol of both insulinomas and normal islet cells. Cytoplasmic expression of p27^kip1^ protein was the predominant localization and was similar in both cases. However, there was a significant difference between the nuclear expression of p27^kip1^, in insulinomas and in normal islet cells (Figure 4). In contrast to normal tissue, where nuclear expression of p27^kip1^ was present in just a few cases (4/24), nearly half of the insulinomas (10/24) showed p27^kip1^ nuclear expression. In seven of them, there was no nuclear staining in their corresponding normal tissue (Wilcoxon signed-rank test, *p* = 0.029).

We also studied the phosphorylated form of p27^kip1^ which presented a different distribution pattern than p27^kip1^. p-p27 (Thr187) showed exclusive cytoplasmic localization in both insulinomas and normal islet cells; interestingly, insulinomas presented higher staining than normal tissues (Wilcoxon signed-rank test *p* = 0.026).

No differences between the subcellular localization of p27^kip1^ and activation of Akt could be observed between malignant and benign insulinomas.

## 4. Discussion

Insulinomas, unlike other pNET, are typically benign tumors, and therefore it is possible to speculate that this might be due to particular molecular events. To the best of our knowledge, this is the first study describing the expression of Akt and p27 proteins in a large series of human insulinomas. In this study, we showed that both total Akt and p-Akt were underexpressed in human insulinomas compared to the normal pancreas islet cells. In spite of this underexpression, the predominant form of Akt was p-Akt. An important regulator of Akt activity is PTEN. PTEN loss of function occurs in a wide spectrum of human cancers through mutations, deletions, transcriptional silencing, or protein instability, resulting in increased activity of the PI3K signaling pathway that leads to elevated levels of phosphorylated AKT [19]. We did not investigate PTEN, but it could be assumed that it would not be useful due to the low expression of p-Akt found in insulinomas.

Our results differ somewhat from previous published evidence showing the activation of the Akt pathway in neuroendocrine tumors [20]. This could be attributed to the inclusion in previous publications of different neuroendocrine tumors, originated not only from the pancreas but also from the gastrointestinal tract and even the lungs, most of them with different clinical behavior [9].

We showed that adult normal beta cells had predominantly cytoplasm p27^kip1^ protein localization. This is in concordance with a previous report showing that most quiescent adult human beta cells present cytoplasmic p27^kip1^ localization, which shifts to nuclear localization during induction of proliferation [21]. This is consistent with our data showing that nearly half of insulinomas present nuclear p27^kip1^ expression, while normal beta cells show nuclear p27^kip1^ expression in only few cases. Protein p27^kip1^ leads to the accumulation and activation of cyclin D/Cdk4 complexes in the nucleus [22], which would initially phosphorylate the Rb protein. This could favor the interaction and subsequent phosphorylation by cyclin E-Cdk2 which leads to hyperphosphorylation and inhibition of the Rb protein [23, 24]. Studies in mice with constitutive activation of Cdk4 showed hyperplasia in their pancreatic islets resembling insulinomas [23]. In addition, the persistence of nuclear p27^kip1^ might also reflect the combination of proliferation while maintaining a differentiated phenotype. As a CDK inhibitor, nuclear p27^kip1^ interferes with CDK4-cyclin D activity and, as a consequence, cell cycles become longer through the extension of the G1 phase. High G1 CDK-cyclin activity, a short G1 phase, or a combination of both, promotes the undifferentiated state of embryonic stem cells. Contrastingly, the increased length of G1 may allow time to respond to external signals and to accumulate differentiation-inducing transcription factors, which could explain the characteristic of insulinomas to keep the ability to secrete insulin as normal differentiated beta cells [25]. Finally, unlike other tumors, cytoplasmic p-p27^kip1^showed no correlation with clinical behavior and no differences were found between benign or malignant insulinomas.

In summary, we could hypothesize that mitogenic signals seem not to activate the Akt pathway in the pathogenesis of human insulinoma. Actually, low expression of Akt might be a disadvantage factor for tumor growth and it might explain the usual benign behavior of insulinomas. Cytoplasmic p27^kip1^ in both insulinomas and normal islet cells may reflect the low rate of replication of beta cells, while nuclear p27^kip1^ would seem to indicate the role of stabilization and nuclear anchoring of the cyclin D-Cdk4 complex. The activation of this complex could allow the reentry of tumor cells from G0 to G1, a marker of proliferative rather than inhibitory status.
